# New Method of Performing Circumcision for Phimosis

**Published:** 1876-07

**Authors:** Jno. Thad. Johnson

**Affiliations:** Professor of Anatomy, and Lecturer on Venereal Diseases, in Atlanta Medical College. Atlanta, Ga.


### NEW METHOD OF PERFORMING CIRCUMCISION FOR PHIMOSIS.

BY JNO. THAD. JOHNSON, M. D.,
Professor of Anatomy, and Lecturer on Venereal Diseases, in Atlanta Medical College.

This operation seems a very simple one, yet many mistakes, at once inconvenient and ludicrous, have occurred in its performance. The cause of such mishap is found in the great elasticity, or extensibility, of the tegumentary covering, and the lack of a corresponding elasticity in the mucous portion of the prepuce. Further, there being no fixed connection between these two layers of the prepuce, their sliding, or rolling, renders it not easy to divide them both at the same point.

The operation that I practice relieves the operator of any fear of this risk. First, I determine the point at which I will remove the prepuce. This is done by marking the integument with nitrate of silver, or indelible ink; or, better, by a nick of the knife, or transfixion with a needle. I then transfix with my bistoury the entire prepuce at this point, and make two flaps of it, by cutting out at its extremity—the flaps, of course, embracing only the portion to be finally removed. Thus we obtain easy access to the inner surface of the redundant prepuce, and have its two layers under perfect control. We now remove the tegumentary layer of one of the flaps at the point previously selected, and then put the mucous layer on the stretch, and remove it at the corresponding point.

The mucous membrane, I repeat, unlike the integument, needs to be put somewhat *on the stretch.* The other flap is then divided in the same manner, on a level with the first. The stump presents the same appearance as when properly made in the more usual manner. The edges of the two layers are then stitched with fine silk sutures. These edges may be more neatly approximated by retracting the whole integument of the penis, and turning back the mucous layer like a cuff.

The operation is much simpler than will be inferred from the complicated description given above. Its only advantage over those generally practiced is a certainty of securing the desired shape and length. It avoids the possible accident of leaving too much mucous membrane for use and too little skin for elegance.

A patient, on whom I recently practiced this operation, presented some points of interest, in that we could observe the effect of a previous operation in which the mistakes above hinted at were made, and then aggravated by leaving the cut edges to take care of themselves without even a stitch to secure them. The skin was still rather long; the mucous membrane was contracted into a complex mass, but when put on the stretch was full two inches in length. The phimosis was still unrelieved. The edges of both layers were ulcerated. It was well-nigh impossible to find the meatus, even with a probe. The patient had been thus treated, or mistreated, in Texas. Coming to this city, he came under care of Dr. H. B. Lee, and with the Doctor I saw him. The method described answered admirably for unraveling the hidden condition, and securing a perfect result.

